# Protective effect of ursodeoxycholic acid upon the post-myocardial infarction heart

**DOI:** 10.1093/cvr/cvaf133

**Published:** 2025-09-12

**Authors:** Benedict Reilly-O’Donnell, Elisa Ferraro, Ryan Brody, Roman Tikhomirov, Catherine Mansfield, Catherine Williamson, Natalia Trayanova, Fu Siong Ng, Julia Gorelik

**Affiliations:** National Heart and Lung Institute, Imperial College London, Hammersmith Campus, ICTEM, 72 Du Cane Road, London W12 0NN, United Kingdom; National Heart and Lung Institute, Imperial College London, Hammersmith Campus, ICTEM, 72 Du Cane Road, London W12 0NN, United Kingdom; Department of Biomedical Engineering, Johns Hopkins University, Baltimore, MD, USA; National Heart and Lung Institute, Imperial College London, Hammersmith Campus, ICTEM, 72 Du Cane Road, London W12 0NN, United Kingdom; National Heart and Lung Institute, Imperial College London, Hammersmith Campus, ICTEM, 72 Du Cane Road, London W12 0NN, United Kingdom; Department of Metabolism, Digestion and Reproduction, Imperial College London, London, United Kingdom; Department of Biomedical Engineering, Johns Hopkins University, Baltimore, MD, USA; National Heart and Lung Institute, Imperial College London, Hammersmith Campus, ICTEM, 72 Du Cane Road, London W12 0NN, United Kingdom; National Heart and Lung Institute, Imperial College London, Hammersmith Campus, ICTEM, 72 Du Cane Road, London W12 0NN, United Kingdom

**Time of primary review: 25 days**

Ursodeoxycholic acid (UDCA) is a secondary bile acid, which has been previously indicated as beneficial in cases of intrahepatic cholestasis of pregnancy and primary biliary cholangitis. There is growing evidence that UDCA may be beneficial in the heart, where it has been shown to have anti-fibrotic^1^ and antiarrhythmic^2^ properties. Recently, Mihajlović *et al*.^3^ have shown that UDCA is cardioprotective in isoprenaline-induced myocardial infarction (MI) rats.^3^ However, longer-term studies on the cardioprotective properties of UDCA upon the post-MI failing heart have not yet been established. Post-MI, extensive ventricular remodelling occurs, which progressively deteriorates contractile function and impairs chamber performance, increasing the likelihood of arrhythmias and heart failure (HF).^4^ The development of HF, after an MI, accounts for a significant number of life-threatening ventricular arrhythmias.^5^ This is due to the arrhythmogenic nature of the post-MI remodelled myocardium.^4^ Our hypothesis was that UDCA protects the post-MI failing heart against dangerous arrhythmia through its dual effect as an anti-fibrotic and anti-arrhythmic agent.

We investigated the effect of UDCA upon a 16-week post-MI rat model. Male Sprague Dawley rats underwent MI surgery (permanent ligation of the left anterior descending coronary artery) or sham surgery, as previously described.^6^ Surgery was performed under anaesthesia (3% isoflurane and 95% Oxygen mix, using a rodent facemask) and 0.05 mgkg^−1^ Buprenorphine (subcutaneous) as analgesia. Bupivacaine (2 mgkg^−1^) was also administered subcutaneously around incision sites. This work was performed in accordance with standards set out in the UK Animals (Scientific Procedures) Act 1986, and approved by Imperial College London Ethical Review Board under the project licence PEE7C76CD. All animal procedures conformed to the guidelines from Directive 2010/63/EU of the European Parliament on the protection of animals used for scientific purposes. Post-surgery, animals were sub-divided into two groups namely; MI and MI + UDCA. With the latter administered 150 mg/kg/day UDCA by gavage (MI received PBS as vehicle control), there was no UDCA-treated sham control group. We combined *in vivo*, *ex vivo* and histological methods to characterize the effect of UDCA upon the animal model. Echocardiograms were performed according to the American Society for Echocardiography leading-edge method^7^ at 0, 8 and 16 weeks post-surgery, under anaesthesia (1.5% isoflurane). After this period, hearts were used for optical mapping and histological studies, animals were anesthetised with 5% isoflurane and 95% Oxygen mix in an induction chamber (until confirmed loss of righting ability and pedal pinch reflex) and then sacrificed by cervical dislocation. Optical mapping studies were performed as previously described.^2^ Propensity to sustained VF induction was scored as previously described.^8^ For histological studies, hearts were fixed in 4% PFA and sectioned into 10 µm slices before staining with Picrosirius Red. Images were acquired using the Zeiss Axio Observer with a 10× objective. Analysis was performed offline with Fiji software, using a custom macro for semi-automated quantification of core infarct and Infarct Border Zone (IBZ).

Computational inducibility experiments were conducted using a three-dimensional human LV ICM model from previous work,^9^ with electrophysiological properties assigned to regions of the virtual heart as previously described.^9,10^ Two features were then modified based upon our findings in the animal model namely: fibrosis distribution and IBZ conduction velocity (CV). ventricular tachycardia (VT) inducibility was tested via an extra-stimulus protocol at seven sites distributed throughout the LV. Electrical propagation simulations were executed by solving the monodomain equations using the software package CARP on a parallel computing system. The inducibility at each pacing location was then compared between simulations.

We found that chronic dietary supplementation of UDCA prevents adverse remodelling of the myocardium, maintaining function so that it was comparable to sham. UDCA attenuated the structural and functional remodelling of the LV in the MI rat heart, when monitored by echocardiogram. At 16 weeks post-surgery the LVEDd, LVESd, area at diastole and area at systole of the MI group were significantly higher than the sham group (*Figure 1A*i, ii, iv and v), this corresponded with a reduction of LV ejection fraction and fractional area shortening (*Figure 1A*iii and vi). UDCA-treated animals showed a significant improvement over the MI group in LVEDd, LVESd and area at systole, but not area at diastole. The UDCA-treated animals were comparable to the sham group in all parameters at 8 and 16 weeks post-MI.

**Figure 1:**
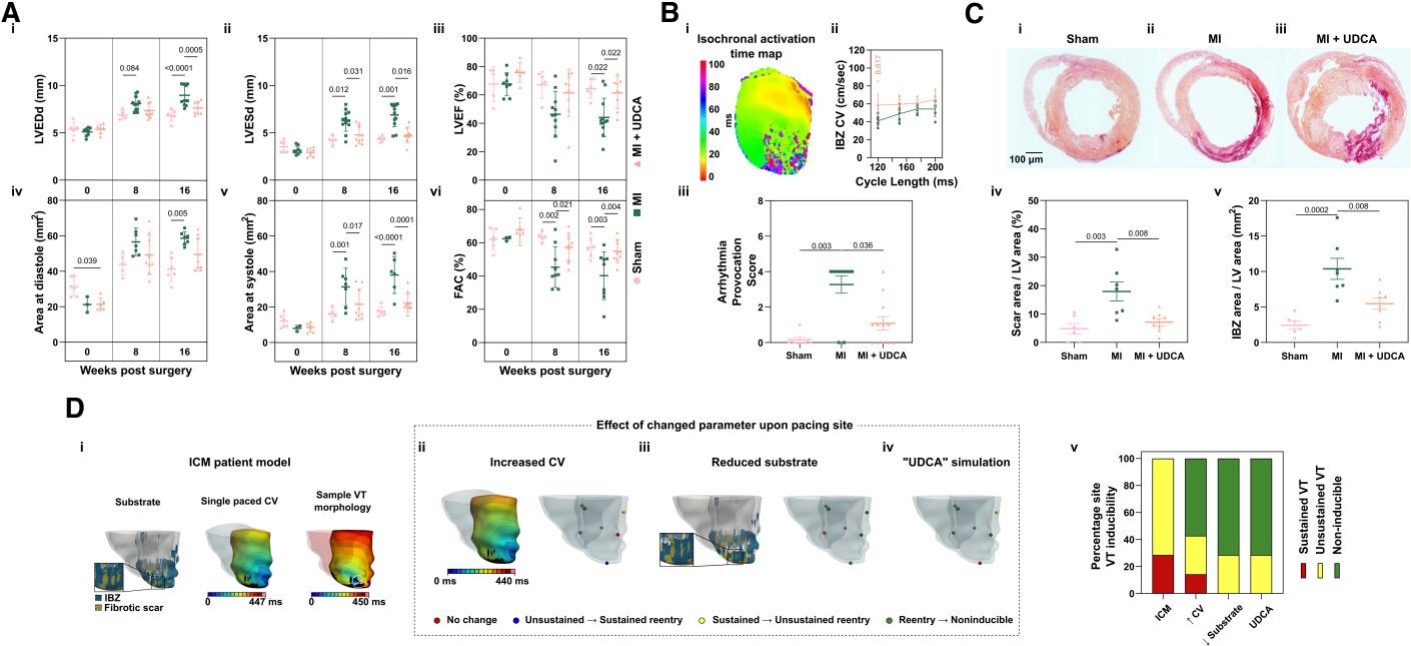
UDCA prevents adverse LV remodeling, improves CV and reduces fibrosis in the IBZ in the post-MI heart. (*A*) Quantitative analysis of echocardiogram from Sham operated (*N* = 5–6), MI (*N* = 3–12) and MI + UDCA (*N* = 6–10) adult rats 0, 8 and 16 weeks post-MI surgery: (*A*i) LVED diameter, (*A*ii) LVES diameter, (*A*iii) LV ejection fraction, (*A*iv) area at diastole, (*A*v) area at systole and (*A*vi) Fractional area shortening. (*B*i) Representative isochronal activation time map of the 16 week post-MI rat heart. (*B*ii) CV from the IBZ, at different pacing cycle lengths (MI: *N* = 6–8, MI + UDCA: *N* = 8–10). (*B*iii) Arrhythmia provocation score of hearts to PES (*N* = 6–12). (C) Representative micrographs showing picrosirius red staining of ventricular tissue at the papillary muscle level in (*C*i) Sham, (*C*ii) MI and (*C*iii) MI + UDCA adult rats. (*C*iv) Summary data of scar area normalized to LV area at papillary muscle level. *N* = 6–8. (*C*v) Summary data of IBZ quantification at papillary muscle level. *N* = 6–8. (D) A human left ventricular ischaemic cardiomyopathy computational model was constructed using an existing patient MRI with late gadolinium contrast. (*D*i) The model included an arrhythmic substrate with IBZ and core fibrotic scar regions. When virtually paced at 7 sites, VT occurred in all simulations (2× sustained, 5× unsustained VT). The model was tested with (*D*ii) increased CV (*D*iii) reduced arrythmic substrate or (*D*iv) a combination of increased CV and reduced substrate, simulating the effect of UDCA. (*E*) Summary of pacing site VT inducibility from all four models tested. Individual data points are plotted along with mean ± SD. Normally distributed values were assessed by one-way or two-way ANOVA followed by Tukey’s multiple comparison. Non-normally distributed data were assessed for statistical significance using the Kruskal-Wallis test, with Dunn’s multiple comparison or Sidak’s multiple comparrisons test. Key *P*-values are indicated on the plots.

In our *ex vivo* optical mapping studies (*Figure 1B*i), we found that UDCA improved CV in the IBZ at short cycle lengths (*Figure 1B*ii). We defined the IBZ according to Rutherford *et al*.^11^ There was no change in action potential duration at any cycle lengths at the IBZ. This faster CV resulted in hearts more resistant to pro-arrhythmic stimuli (*Figure 1B*iii). Programmed electrical stimulation (PES) to provoke re-entrant arrhythmias generated VT in 9/11 MI hearts (APS = 4.27 ± 1.62), vs. 7/12 hearts in MI + UDCA (APS = 2.08 ± 1.31, *P* = 0.036).

Histological staining at the papillary muscle level (approximate position of the IBZ, *Figure 1C*i–iii), showed that scar area was significantly increased in the post-MI heart when compared with sham; however, in MI + UDCA hearts, collagen staining was significantly reduced when compared with MI (5.46 ± 2.21, vs. 10.37 ± 3.93%, *P* = 0.008, *Figure 1C*iv), This pattern was also observed at the IBZ, where area was increased in the post-MI heart but not in the UDCA-treated heart (*Figure 1C*v).

To assess the potential translational impact of UDCA treatment, computational simulations were run on a human LV model with a patient-specific ischaemic substrate generated from late- gadolinium enhanced cardiac MRI (*Figure 1D*i). A virtual PES protocol was applied to four scenarios: HF (control), increased CV (*Figure 1D*ii), reduced substrate (scar) area (*Figure 1D*iii) and substrate reduction in combination with increased CV, representing the UDCA- treated heart (*Figure 1D*iv). The parameters of the UDCA simulation were based upon our ex vivo data (*Figure 1B* and *C*). The model with increased CV was non-inducible at 4 out of the 7 originally inducible pacing sites, while the model with reduced substrate volume was non-inducible at 5 of 7 sites. When both effects were combined to simulate the effect of UDCA, 5 of 7 sites were non-inducible (*Figure 1D*v).

This study provides evidence that administration of UDCA in a rat model of chronic HF prevents the adverse LV remodelling associated with the progression of MI and reduces fibrosis and the healed IBZ. This is in agreement with our previous study of the effect of UDCA upon human fibroblasts and living myocardial slices.^1^ At 16 weeks post-MI, hearts had a reduced susceptibility to ventricular arrhythmias at PES and improved CV across the IBZ in when treated with UDCA, we suggest this may be due to preserved phosphorylation of connexin-43, as has been previously observed.^2^ Transferring our experimental data to an *in silico* 3D human LV model, we found that a combination of reduced arrhythmic substrate area and improved CV (two identified benefits of UDCA-treated post-MI rats) acted together to reduce arrhythmogenicity.

This study identifies that UDCA is anti-fibrotic and anti-arrhythmic in post-MI rat. We found that simulation of UDCA treatment on a human model reduced the occurrence of VT. This indicates that UDCA is a candidate treatment for the prevention of post-MI HF (& arrhythmia) through anti-fibrotic and anti-arrhythmic effects.

## Authors’ contributions

B.R.-O. wrote the manuscript. E.F. performed the experiments. E.F., B.R.-O. and R.T. performed data analysis. R.B. and N.T. produced the left ventricular computational model. C.M. performed MI surgery. B.R.-O., E.F., J.G. and F.S.N. designed experiments and conceived the project. J.G., C.W. and F.S.N. supervised the project. All authors reviewed the manuscript.

## Data Availability

The data underlying this article are available in the article and upon request.
